# The Effects of Resveratrol on Prostate Cancer through Targeting the Tumor Microenvironment

**DOI:** 10.3390/jox11010002

**Published:** 2021-02-01

**Authors:** Natalie Silk, Jeremy Reich, Rahul Sinha, Shivansh Chawla, Kyla Geary, Dianzheng Zhang

**Affiliations:** Department of Bio-Medical Sciences, Philadelphia College of Osteopathic Medicine, Philadelphia, PA 19131, USA; ns9703@pcom.edu (N.S.); jr9109@pcom.edu (J.R.); rs8771@pcom.edu (R.S.); sc8873@pcom.edu (S.C.); kylage@pcom.edu (K.G.)

**Keywords:** resveratrol, prostate cancer, tumor microenvironment, stroma

## Abstract

Prostate cancer is one of the most common cancers diagnosed in men in the United States and the second leading cause of cancer-related deaths worldwide. Since over 60% of prostate cancer cases occur in men over 65 years of age, and this population will increase steadily in the coming years, prostate cancer will be a major cancer-related burden in the foreseeable future. Accumulating data from more recent research suggest that the tumor microenvironment (TME) plays a previously unrecognized role in every stage of cancer development, including initiation, proliferation, and metastasis. Prostate cancer is not only diagnosed in the late stages of life, but also progresses relatively slowly. This makes prostate cancer an ideal model system for exploring the potential of natural products as cancer prevention and/or treatment reagents because they usually act relatively slowly compared to most synthetic drugs. Resveratrol (RSV) is a naturally occurring stilbenoid and possesses strong anti-cancer properties with few adverse effects. Accumulating data from both in vitro and in vivo experiments indicate that RSV can interfere with prostate cancer initiation and progression by targeting the TME. Therefore, this review is aimed to summarize the recent advancement in RSV-inhibited prostate cancer initiation, proliferation, and metastasis as well as the underlying molecular mechanisms, with particular emphasis on the effect of RSV on TME. This will not only better our understanding of prostate cancer TMEs, but also pave the way for the development of RSV as a potential reagent for prostate cancer prevention and/or therapy.

## 1. Introduction

For men, prostate cancer is the second most common cancer in the world, resulting in over 350,000 deaths in 2018 [1]. Most prostate cancers are adenocarcinoma, originating from epithelial cells in the peripheral zone of the prostate [2]. The survival rate for patients with prostate cancer confined to the primary site is nearly 100% [3]. If left unchecked, prostate cancer progresses and eventually metastasizes to form new cancers in other tissues throughout the body. The overwhelming majority of prostate cancer–related deaths occur due to metastasis, most often to the bone [4]. Since prostate cancer incidence increases exponentially for men over 65 years, and this population will increase dramatically in the coming years, prostate cancer is expected to be a big burden both socially and economically in the foreseeable future [1].

Because of the importance of the androgen/androgen receptor (AR) signaling pathway in prostate cancer initiation and progression [5], most basic and clinical research has been focused on the androgen/AR axis. Androgens such as testosterone and dihydrotestosterone bind AR in the cytoplasm and the ligand-bound AR is translocated to the nucleus to interact with AR responsive elements (ARE) and transcriptionally regulate the expression of AR target genes such as prostate-specific antigen (PSA) [6]. Dysregulation of the androgen/AR axis can lead to cancer cell proliferation, escaping from apoptosis, and metastasis [7,8]. Multiple lines of evidence from recent research indicate that, in addition to the androgen/AR axis, the tumor microenvironment (TME) plays an indispensable role in prostate cancer initiation and progression [9,10]. The TME is composed of fibroblasts, the extracellular matrix (ECM), immune cells, and other factors in the tumor cell’s surroundings [11]. There is constant communication between tumor cells and the TME [12]. Fibroblasts, especially cancer-associated fibroblasts (CAFs), play important roles in each of the developmental stages of different cancers, including prostate cancer [9,13]. In fact, AR signaling is not only involved but also plays an important role in TME [14,15], although the extent of and exact mechanisms involved in AR-mediated changes in TME are still being elucidated.

Compared to most other cancers, prostate cancer progression is usually much slower, so the most common approaches taken with early and low-risk prostate cancers are non-invasive active surveillance and watchful waiting [16]. Advanced prostate cancer is treated with radical prostatectomy, radiotherapy, chemotherapy, and androgen-deprivation therapy (ADT) through physical or chemical castration to repress androgen-induced AR signaling [17]. Although responsive to ADT at the beginning, most prostate cancers become ADT-resistant over time [18] due to the formation of constitutively active AR variants or the activation of AR by other factors [9]. For castration-resistant prostate cancer (CRPC) [19], treatment is usually the combination of traditional ADT and second-line antiandrogen drugs such as abiraterone acetate, an androgen synthesis inhibitor, or enzalutamide, to inhibit AR directly [20,21]. Accumulating data demonstrate that resveratrol (RSV), a stilbenoid produced in multiple plants, possesses strong anti-cancer properties [22,23]. By targeting both AR [24] and the TME [25,26], RSV can induce growth inhibition, cell cycle arrest, apoptosis, as well as inhibit metastasis of different cancer types, including prostate cancer [22,27]. More importantly, since RSV has few side effects at therapeutic concentrations, it can be used as a treatment regimen without fear of complications [28]. To pave the road for the development of RSV as a preventive and/or therapeutic reagent, either by itself or in combination with other drugs, our review focuses on the effects of RSV on the TME in prostate cancer initiation, proliferation, and metastasis.

## 2. RSV Inhibits Prostate Cancer Initiation by Targeting the TME

The exact point at which prostate tissue transitions from normal to cancerous as well as the underlying molecular mechanisms remain ambiguous. However, multiple factors, including the TME, play important roles in the initiation of tumors, including those in the prostate. Although different components of the TME have their roles in cancer development, the communication between the prostate stromal and epithelial cells plays an indispensable role in prostate cancer initiation. Results from research using a mouse model to study cancer initiation in submandibular glands indicate that the interaction between stroma and epithelium is essential because malignancy only occurred when stromal and epithelial cells grew together [29]. Following suit, Chung et al. demonstrated the essentiality of stromal cells in prostate cancer initiation [30]. Since transitional carcinoma cells from mice bladder can grow together with embryonic urogenital sinus mesenchyme (UGM), UGM was used to mimic the reactive human prostate stroma to induce prostatic neoplasms. These results indicate that crosstalk between the stromal and epithelial cells not only exists but also is imperative for the initiation of prostate neoplasia. However, since humans have a much higher proportion of smooth muscle and overall stromal cells than mice [31], this difference needs to be taken into consideration for any solid conclusions about the role of stroma in prostate cancer initiation. Therefore, similar studies in humans will be an important step for an accurate depiction of reactive stroma in prostate cancer initiation.

The development of prostate cancer is very similar to the development of benign prostate hyperplasia (BPH) because they share similarities at the molecular, cellular, genetic, hormonal, and inflammatory levels [32]. Therefore, studying the initiation of benign prostate hyperplasia may also provide insight into prostate cancer initiation. Hayward et al. performed a tissue recombination study by co-culturing benign prostate epithelial cells with stromal cells from either BPH or normal prostate tissues [33] and found that significant epithelial proliferation occurred only when co-cultured with BPH stromal cell lines, indicating that stromal cells have an important role in BPH development. It is important to note that although stromal cells can influence the development of malignancy [34], the communication between the stroma and epithelium is bidirectional. Epithelium-derived cancer cells are capable of influencing the stroma, specifically that of the smooth muscle. Co-culturing of 18-day embryonic rat UGM tissue with either normal epithelial cells or neoplastic prostate epithelial cells showed that the cells from the normal epithelia induce ductal structure formation in smooth muscle, while the cells from neoplastic prostate epithelia failed to do so [33]. These findings altogether indicate that the stromal cells play an important role in prostate cancer initiation, and malignant cells are capable of affecting stromal cell behavior. Given the importance of stroma–epithelium crosstalk in prostate cancer initiation, interrupting this communication could be a plausible strategy in the prevention of prostate cancer initiation [35].

**Figure 1:**
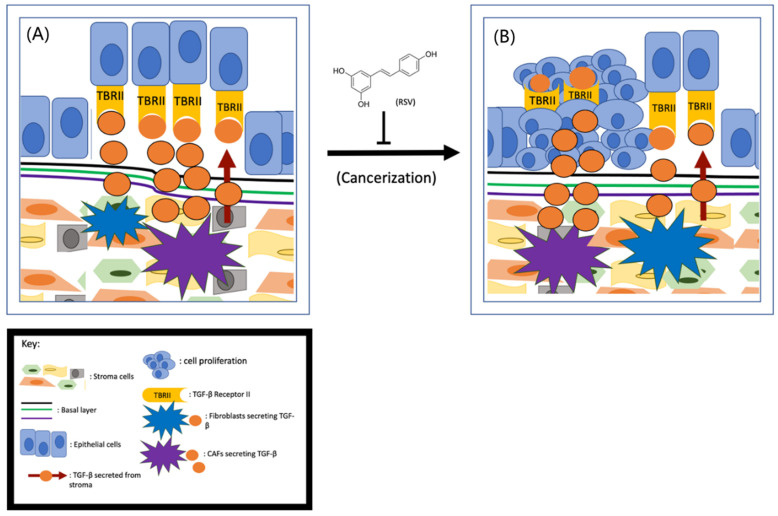
RSV inhibits prostate cancer initiation (**A**) CAFs- secreted TGF-β interacts with its receptors TBRII on prostate cells drives tumor initiation (**B**) and RSV is capable of inhibiting this process.

Although multiple lines of evidence indicate that stroma–epithelium crosstalk is essential in the initiation of prostate cancer, the exact role of stromal cells in prostate cancer initiation is currently unknown. Based on the fact that the stromal cells in non-cancerous tissues are more stable than in cancerous tissues, it has been postulated that it is the cancer-associated fibroblasts (CAFs), not the normal stromal cells, that induce tumor initiation [35]. Cunah et al. engrafted BPH-1 cells with either normal stroma or CAFs to adult male mice and found that tumors formed only when the cells were co-engrafted with CAFs [36]. Therefore, it is hypothesized that the CAFs may secrete excess growth factors and/or cytokines to protect the tumor cells from apoptotic death and subsequently lead to tumor formation [37]. As depicted in Figure 1, CAFs secrete more transforming growth factor-beta (TGF-β) than the normal stroma [38]. By activating TGF-β Receptor II (TBRII), the TGF-β can enhance cell proliferation, differentiation, and extracellular matrix production. Given that the prostate epithelial cells are TGF-β Receptor II (TBRII)-positive [39], it is fully conceivable that TGF-β/TBRII can mediate the communication between CAFs and prostate epithelial cells (Figure 1).

Since the cancer-preventive effects of RSV were first reported in 1997 [22], both epidemiological and case-controlled studies have demonstrated that RSV and/or consumption of foods/drinks with high levels of RSV can reduce prostate cancer incidence [40]. These findings suggest that RSV could potentially inhibit prostate cancer initiation. Based on the role of CAFs in prostate cancer initiation, early research was focused on the effect of RSV on CAFs. To determine if RSV can inhibit CAF-induced prostate cancer initiation, Wen et al. examined the effect of RSV on TGF-β secretion and found that RSV is capable of downregulating TGF-β expression from CAFs [8]. Further research demonstrated that the downregulation of TGF-β is partially due to RSV’s repressive effect on the Wnt/Beta-catenin pathway [41]. Given that the Wnt pathway in prostate epithelial cells can be activated by CAFs, and RSV is capable of inhibiting TGF-β secretion from CAFs, RSV is likely to inhibit prostate cancer initiation by targeting the TGF-β/TBRII axis. Also, activation of the JAK2/pSTAT1 pathway activates mast cells, leading to inflammation and the transition of stroma to CAFs [42]. Since RSV can cause mast cells to reduce fibrosis by targeting the SCF and c-kit pathway [43], it is also likely that RSV inhibits prostate cancer initiation through repression of mast cell–mediated stromal cell transition to CAFs (Figure 1). However, to demonstrate that RSV can inhibit prostate cancer initiation unambiguously, it is important to conduct both in vitro and in vivo experiments to demonstrate RSV’s role in inhibiting prostate cell cancerization.

## 3. RSV Inhibits Prostate Cancer Cell Proliferation by Targeting the TME

Two of the most fundamental traits of cancer cells are their abilities to proliferate without regulation and evade cell death [44], and both of these traits can be mediated by the TME. As described in the previous section, crosstalk between the stroma and epithelium under tumorigenic conditions is a key driver in prostate cancer growth [44,45]. The normal fibroblasts acquire a modified phenotype (phenoconversion) and become perpetually activated CAFs [46,47]. The normal fibroblasts secrete only enough factors to maintain normal tissue function and have a relatively low proliferative potential [48]. But the increased secretion of growth factors and cytokines by the CAFs [49] exposes the tumor cells to an altered TME favoring cell growth and proliferation. Therefore, the transition of pre-carcinogenic stroma to CAFs is a critical mechanism for prostate cancer cell proliferation and cancer progression.

### 3.1. RSV Inhibits Prostate Cancer Proliferation by Interrupting Stroma–Tumor Cell Communication

It has been shown that RSV can affect the proliferation of prostate cancer cells both in vitro and in vivo. For example, treating androgen-responsive human prostate cancer cells (LNCaP) with different concentrations of RSV demonstrated that RSV can inhibit cancer cell proliferation in a concentration-dependent manner. One possible explanation is that RSV targets the AR- and ER-dependent signaling pathways because RSV is capable of counteracting both androgen- and estrogen-induced cell growth [50]. RSV also can inhibit prostate cancer cell proliferation in both transgenic rat adenocarcinoma of prostate (TRAMP) mouse and tumor xenograft models. Harper et al. showed that when transgenic mice were fed a diet containing RSV, cell proliferation in the dorsolateral prostate and ventral prostate decreased significantly [51]. The authors also observed that the transgenic mice had a marked increase (42–62%) of well-differentiated prostate tumors compared to the control animals, suggesting that RSV can halt tumor progression. Wang, et al. injected LNCaP cells into mice on an RSV-enriched diet and found that RSV can delay tumor growth. Of note, both Harper and Wang’s studies suggest that RSV targeted the hormone-based signaling pathways [50,51]. However, the RSV-mediated delay was only temporary, as the tumor volumes of the xenograft mice fed RSV eventually caught up with those of the controls. The underlying mechanisms remain unknown.

**Figure 2:**
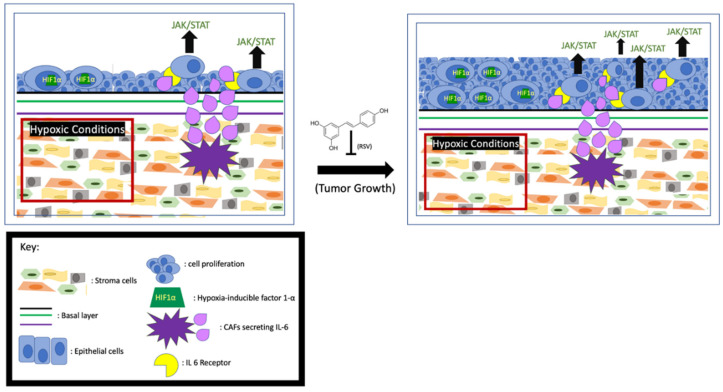
RSV inhibits prostate cancer proliferation. Hypoxia-induced HIF1α and activation of JAK/STAT by the CAFs- secreted IL-6 enhance tumor growth, and RSV is capable of inhibiting cell proliferation.

Increased levels of interleukin-6 (IL-6) have been linked to poor survival among prostate cancer patients, and it is known that CAFs secrete high levels of IL-6 into the TME. One of the signaling pathways activated by IL-6 is JAK/STAT3. Activation of this pathway in the cells leads to tumor cell proliferation and escape from apoptosis. Mechanistically, the IL-6-activated JAK leads to STAT3 dimerization and translocation to the nucleus to upregulate the expression of proliferative proteins like Bcl-x and Mcl-1 [52,53]. These anti-apoptotic proteins collectively (Figure 2) prevent cell death by reducing mitochondrial outer membrane permeability [54]. When LNCAP and 22RV1 cells were treated with IL-6, the STAT3 was phosphorylated and activated [55], accompanied by enhanced cell proliferation. Consistently, the activity of the IL-6/STAT3 signaling pathway is proportional to the growth rate of advanced prostate cancer [56]. Additionally, it has been found that in LNCAP cells the IL-6-activated JAK/STAT3 pathway also downregulates p53, a major cell cycle regulator [57,58]. Multiple lines of evidence indicate that the CAF-secreted IL-6 may also influence tumor growth by affecting AR activity. Hobisch et al. first observed that in prostate cancer cells IL-6 can activate AR in a dose-dependent manner [59]. Soon after, it was found that in LNCaP cells the IL-6-upregulated serine-threonine kinase Pim1 can phosphorylate (serine-213) and activate AR [60,61]. In addition, Kemskova et al. demonstrated that Pim1 is also regulated by STAT3. These findings altogether indicate that the IL-6/JAK/STAT3 pathway can enhance prostate cancer cell proliferation in an AR-dependent manner [62] (Figure 2). Therefore, monoclonal and immunotherapeutic treatments targeting the IL-6/JAK/STAT3 pathway are currently under vigorous investigation [55,63].

It has been reported that RSV can exert a strong anti-proliferative and pro-apoptosis effect on the CAFs in both prostate and lung cancer cell lines [64]. Meanwhile, RSV blunts and even reverses phenoconversion from normal prostate fibroblast to myofibroblast. RSV also indirectly affects IL-6 signaling by inhibiting its downstream factor STAT3 to mitigate proliferative effects. For example, experimental results from mouse fibroblast (NIH3T3) cells and LNCaP cells showed the inhibitory effect of RSV on Src tyrosine kinase, an activator of STAT3, repressing the STAT3 signaling pathway [65,66] and subsequently reducing cell growth and increasing apoptosis (Figure 2). The authors posit that apoptosis is triggered by downregulating downstream factors like Bcl-x, Mcl-, and Cyclin D1 [67,68]. Furthermore, Lee et al. demonstrated that inhibiting IL-6 RSV can repress STAT3-upregulated AR and cell proliferation in prostate cancer cells [25].

### 3.2. RSV Inhibits Cancer Cell Proliferation by Affecting Hypoxic Conditions of the TME

It is well established that aerobic glycolysis, also known as the Warburg effect, occurs in cells of solid tumors. Hypoxia and high acidity are the two main characteristics of the Warburg effect, and both of them favor the proliferation of cells in solid cancer [69]. Numerous signaling pathways in prostate cancer cells respond to the hypoxic TME, and these pathways collectively lead to increased angiogenesis, survival, proliferation, and/or metastasis [70]. Hypoxia-inducible factor 1-α (HIF1α) plays a central role in cellular adaptation to hypoxic conditions. In hypoxia, the stabilized HIF1α transcriptionally regulates the pro-proliferative and anti-apoptotic genes [71]. Meanwhile, O_2_ is more frequently reduced to reactive oxygen species (ROS), such as superoxide (O_2_^−^) [72,73], which can further stabilize HIF1α [74,75]. Understanding the mechanisms by which tumor cells proliferate in response to the hypoxic TME may prove advantageous for targeted inhibition of cancer cell proliferation.

RSV appears to be both pro- and anti-oxidant, depending on the circumstances [76]. In non-cancer tissues, RSV serves as an antioxidant [77], and therefore RSV can exert a beneficial effect on a wide variety of issues, including neuronal [78], anti-inflammatory [79], and cardiac [80]. However, to cancer cells with low pH environments due to the Warburg Effect, RSV shows more pro-oxidant characteristics. Shamim et al. found that RSV can induce cancer cell death by inducing ROS accumulation, which subsequently leads to oxidative DNA damage and apoptosis [81,82,83]. Using the TRAMP model, Wang et al. demonstrated that RSV-enhanced cancer cell death is due to the upregulation of HIF1α, which enhances ROS concentration in the TME beyond the limit for survival [84]. Since RSV preferentially increases superoxide over other ROS species like hydrogen peroxide [85], Low et al. expounded on the pro-apoptotic effects of elevated superoxide levels in the mitochondria [86] and found that superoxide can activate caspases 9 and 3, and subsequently promote the release of cytochrome C and apoptosis (Figure 2). It is important to note that low concentration of RSV can serve as a pro-oxidant that favors cell survival, and pro-apoptotic effects occur only at relatively higher RSV concentrations to stimulate superoxide production. The bidirectional nature of RSV needs to be considered when RSV is used as a therapeutic reagent.

## 4. RSV Inhibits Prostate Cancer Metastasis by Targeting the TME

The migration of cells in solid cancers toward their surrounding tissues is the first step of metastasis. Migratory cancer cells undergo both molecular and cellular changes in response to their TME. These changes are collectively known as epithelial-mesenchymal transition (EMT), in which the epithelial cells lose their cell–cell adhesion and gain migratory and invasive properties. Generally, the stroma in non-cancer tissues is rather stable. However, both genetic and epigenetic changes have been found in CAFs [87]. Given that constant communication occurs between stromal cells and cancerous epithelial cells [13], it is conceivable that CAFs could affect the migratory properties of the cancer cells. Multiple lines of evidence indicate that the CAFs are capable of promoting EMT [30,87] and consequently enhance cancer cell migration and invasion [88,89]. Therefore, understanding stroma-mediated EMT is of importance not only in understanding the underlying molecular mechanism but also for identifying potential agents to prevent or even reverse metastasis [89]. 

### 4.1. Resveratrol Interrupts the Communication between Stromal and Cancer Cells

**Figure 3:**
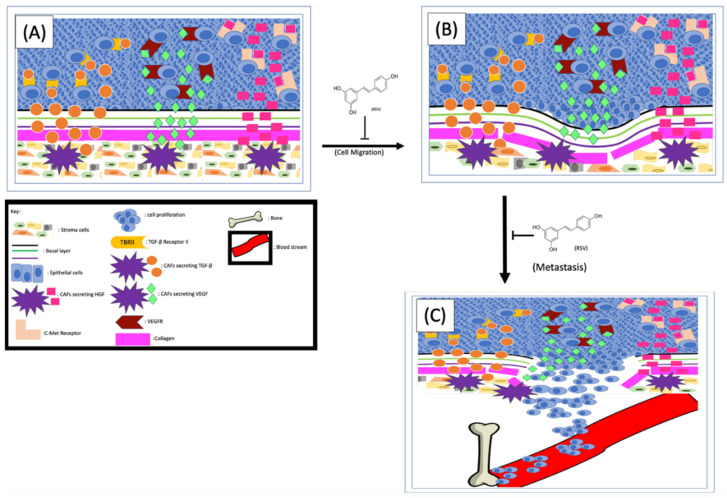
RSV inhibits EMT and cancer cell metastasis. The CAFs-released TGF-β, VEGF and HGF (**A**) interact with their corresponding receptors on cancer cells and enhance EMT processes (**B**). The cancer cells metastasize from their primary site (**B**) to the bone (**C**). RSV is capable of inhibiting prostate cancer metastasis by repressing EMT and cancer cell migration/invasion.

One of the effects that CAFs exerted on cancer cells to enhance EMT is to secret different factors into the TME, which are subsequently sensed by cancer cells [90]. Indeed, the level of hepatocyte growth factor (HGF) in the TME of metastatic prostate cancer is higher than that of non-metastatic cancers [90]. Glenn A. Gmyrek et al. [91] have experimentally demonstrated that HGF is capable of triggering pro-migratory phosphorylation of prostate cancer cells and enhancing migration and metastasis. The same group of researchers further demonstrated that the cultured stromal cells showed a myofibroblastic phenotype and speculated that the myofibroblastic subpopulation of prostate stromal cells could be the source of HGF in vivo [91]. Further analysis found that in prostate tissues the HGF is expressed in the stromal cells but not the epithelium, suggesting the HGF in the TME could be the result of paracrine secretion. Since CAFs secrete higher levels of HGF than normal stromal cells, the CAFs were postulated as the culprits driving cancer cell migration [26,91]. Later, it was experimentally demonstrated that stromal cells are capable of enhancing prostate cancer cell migration and that this effect is HGF-dependent [92]. Mechanistically, the CAF-derived HGF interacts with c-Met receptors on cancer cells to activate numerous signaling pathways, especially those related to EMT and metastasis [90] (Figure 3). Therefore, any strategies interrupting the HGF/c-Met interaction by either inhibiting the secretion of HGF or repressing the cancer cell responses to HGF could inhibit EMT and metastasis. To determine if RSV can repress stromal cell–mediated prostate cancer migration, Hsieh TC et al. co-cultured stromal and cancer cells in the presence or absence of RSV and found that RSV can indeed inhibit stromal cell–enhanced cancer cell migration. Since treatment of the co-culture with either RSV or antibodies specifically against HGF abolished stroma-enhanced migration to a similar degree, the authors proposed that RSV inhibits cancer cell migration by counteracting the stromal cell–derived HGF [92]. These findings altogether indicate that (1) stromal cell–secreted HGF plays important roles in prostate cancer migration and invasion, and (2) RSV inhibits prostate cancer migration at least partially by counteracting HGF (Figure 3).

It is well known that the ion channel transient receptor potential ankyrin 1 (TRPA1) on CAFs is one of the main control mechanisms for HGF secretion. To further understand the mechanism of RSV-inhibited cancer migration, both mouse [93] and rat [94] models were used, and the results demonstrated that RSV can block HGF secretion by inhibiting TRPA1. Additionally, Vancauwenberghe et al. [26] further demonstrated that by inhibiting the TRPA1, RSV is capable of blocking the secretion of both HGF and vascular endothelial growth factor (VEGF), an important factor involving the angiogenesis of tumor tissues. These findings altogether suggest that RSV could be potentially used as a reagent to inhibit prostate cancer metastasis (Figure 3). Of note, this same study [89] also noticed that RSV can exert an opposite effect on cells expressing a mutant TRPA1, which not only increased expression and secretion of HGF and VEGF, but also enhanced prostate cancer metastasis. Therefore, it is important to stratify prostate cancer patients based on their TRPA1 types and apply RSV only to those with wild-type TRPA1. 

### 4.2. Resveratrol Inhibits Prostate Cancer Metastasis by Targeting the Extracellular Matrix

The extracellular matrix (ECM) is a network formed between cells composed of non-cellular components such as protein, glycosaminoglycan, and glycoconjugate. The ECM can provide both structural and biochemical support to the resident cells, and therefore the ECM can affect cellular behaviors. One of the largest components of the ECM is collagen, which is synthesized and secreted by fibroblasts located in the stroma [95]. Communication between collagen and cancer cells exists in every cancer development stage [96]. Additionally, cadherin plays important structural and signaling roles in cell–cell and cell–ECM interactions [97]. E-cadherin usually maintains the integrity of epithelial tissue, and N-cadherin mediates the contact between cells and the matrix. During the EMT process, N-cadherin is usually upregulated with concurrent downregulation of E-cadherin [98]. Combined dysregulations make the tumor cells more motile and favor metastasis, thereby allowing cancer cells to escape from the site of origin. Therefore, the ECM is an additional barrier to break before the cancer cells can escape from the TME.

Activation of the PI3K signaling pathway can lead to collagen production and fibrosis [99]. The transforming growth factor beta-1 (TGFβ-1), secreted from human prostate stromal cells, can reactivate the stroma by stimulating the phenotypic switch of fibroblasts to myofibroblasts, thus further increasing collagen secretion [95]. It has been shown that RSV can inhibit both the TGFβ and PI3K signaling pathways, thereby decreasing the production and secretion of collagen from the stroma. Since collagen is so prevalent in both benign and malignant tissue, it has been shown that, in organs with highly collagenous reactive stroma, the structural properties of collagen contribute to tumor invasion and metastasis [99]. In normal tissue, the collagen production and assembly are regulated by the counterbalance of matrix metalloproteinase (MMP), MMP inhibitors, and other enzymes. The collagen in TME affects tumor cells’ proliferation, differentiation, gene expression, migration, invasion, and metastasis. Since the MMPs in TME are more prevalent, they degrade and remodel the basement membrane, which favors tumor cell migration and metastasis [100]. The inhibitory effects of RSV on TGFβ and/or CXCL12-mediated myofibroblast phenoconversion of prostate fibroblasts in vitro [64] in a concentration-dependent manner have been reported. Since phenoconversion is necessary for increased collagen production [101], and increased collagen has been shown to contribute to tumor invasion and metastasis, it is conceivable that RSV might be able to inhibit prostate cell metastasis by preventing fibroblast to myofibroblast transition (Figure 3).

The stromal-cell–secreted MMPs play an indispensable role in cancer cell metastasis, with MMP-2 and MMP-9 being the best-studied MMPs in prostate cancer metastasis. In tissues with non-migrating cells, the MMPs are inhibited by tissue inhibitors of metalloproteinase (TIMPs). However, upregulated expressions of MMP-2 and MMP-9, accompanied by downregulated TIMPs, were found in high-grade tumors [102], suggesting that interrupting the delicate balance between MMPs and TIMPs could affect tumor cell migration and cancer metastasis. Once secreted, both MMP-2 and MMP-9 promote metastasis by either enhancing the degradation of barriers of the ECM, such as collagen and laminins, or affecting the balance between cell–cell and cell–matrix attachments [103]. Since the inhibitory effect of RSV on MMPs has been shown in many cancer types, and RSV is capable of inhibiting both MMP-2 and MMP-9 [102], the inhibitory effect of RSV on prostate cancer was examined in different models. The results from the animal models indicate that RSV and the tumor necrosis factor (TNF)-related apoptosis-inducing ligand (TRAIL) can act individually; the combination of them inhibits prostate cancer metastasis synergistically [104]. Also, it has been noted that androgen treatment of the LNCaP cells can morphologically alter the cells from spindle-like into fibroblast mesenchymal cell shape, and RSV is capable of inhibiting the androgen-induced morphological change [105]. Since this kind of morphological change usually occurs during the EMT process, it further implies that RSV may play a role in prostate cancer cell EMT. In addition, it appears that RSV is also capable of regulating the expression of cadherin. Loh et al. showed that RSV can restore the epithelial phenotype of the mesenchymal cells and inhibit the expression of EMT-related markers [106]. Consistently, our unpublished data also support the notion that RSV can inhibit EMT by up- and downregulating E-cadherin and N-cadherin, respectively, in prostate cancer cells.

### 4.3. Inhibitory Effects of RSV on Prostate Cancer Cell Bone Metastasis

Once escaped from their primary sites into the bloodstream, cancer cells can travel through the blood and resettle in different organs of the body, although prostate cancer cells preferentially metastasize to the bone. Therefore, metastatic bone cancer is the leading cause of death for patients with prostate cancer [4]. A better understanding of the communication between prostate cancer cells and the bone microenvironment is the first step in designing strategies to prevent bone metastasis of prostate cancer [107].

The CXCR4/CXCL12 axis plays essential roles in all stages of prostate cancer progression [108], including bone metastasis [109]. Interaction between CXCL12 and CXCR4 leads to the activation of multiple downstream pathways such as MAPK and JNK [110] and ultimately causes cytoskeletal rearrangements, actin polymerization, and integrin-dependent adhesion to endothelial cells [110]. On the one hand, higher levels of CXCL12 are expressed in bone marrow stromal cells [109]. On the other hand, most prostate cancer cells express elevated levels of CXCR4, which makes the CXCL12/CXCR4 axis more active in these cells. Indeed, CXCL12 can enhance the adhesion of prostate cancer cells with osteosarcoma cells [111]. In addition, Sun et al. compared the levels of CXCL12 in different tissues and found that, in both human and mouse models, the levels of CXCL12 are higher in the prostate cancer–preferred metastatic tissues than in those rarely targeted [112]. To further demonstrate how essential CXCR4 is to prostate cancer bone metastasis, the authors neutralized the receptor by injecting antibodies against CXCR4 and found that prostate-cancer-to-bone metastases were significantly inhibited [112]. 

Compared to the surrounding non-cancerous tissues, the vascular endothelial growth factor (VEGF) is upregulated in most solid cancers, including prostate cancer, and one of the major sources of VEGF in the TME is CAFs [113]. Consistently, overexpression of VEGF receptors (VEGFR) has been found in cancerous endothelial cells, which play an indispensable role in cancer cell migration and metastasis [114]. More recent results indicate that the interaction between the tumor and stromal cells can further induce HIF1α-mediated VEGF secretion from the CAFs [38,115], which could be part of the reason why elevated levels of VEGF are found in cancerous tissues but not the benign tissues [116]. One of the effects of VEGF is to support tumor neovascularization through the secretion of proteolytic enzymes to dismantle the ECM, which favors EMT [116]. The precise mechanism for VEGF-mediated prostate cancer metastasis is not yet completely understood. But it has been hypothesized that the bone stroma-secreted VEGF interacts with cancer cells’ VEGFR and subsequently enhances cancer cells nesting to the bone [116]. This hypothesis is supported by the fact that elevated levels of VEGFR-2 were consistently found on prostate cancer cells [117]. More importantly, both VEGF and VEGFR-2 are notably higher at the sites of bone metastasis than at the primary prostate tumors of the same individual, further demonstrating the importance of the VEGF/VEGFR-2 axis in prostate cancer bone metastasis [117]. Interestingly, RSV and its analog HS-1793 are capable of inhibiting both hypoxia-induced VEGF expression and prostate cancer cell migration [115] (Figure 3). In addition, RSV, either alone or in combination with tumor necrosis factor-related apoptosis-inducing ligand (TRAIL), can inhibit the VEGF/VEGFR2 axis in vivo [104]. RSV’s inhibitory effect in bone stroma–prostate cancer cell communication is an ongoing research topic [116,117,118]. 

### 4.4. RSV Inhibits Immune Cell-Mediated Prostate Cancer Metastasis

Although the origin of tumor-associated macrophages is not completely known, different immune cells, especially the macrophages, are found ubiquitously in solid tumors. One school of thought is that they are tissue-resident macrophages, and another postulates that the tumor macrophages are derived from monocytes [80,119]. Nevertheless, it is widely accepted that immune cells not only exist in the TME but also affect the development of prostate cancer, and multiple lines of evidence indicate that RSV is capable of inhibiting the immune cell–mediated prostate cancer metastasis. Based on the number of macrophages found in prostate cancer tissues derived from different patients, Lewis and colleagues concluded that macrophages could lead to either favorable or unfavorable prognosis in prostate cancer [120]. Later, it was found that higher tumor-associated macrophage (TAM) count is correlated with a reduced survival time but an increased disease-free survival after treatment [121,122]. The authors attribute this inconsistency to not only the methodological differences between studies but also the complex functions of macrophages.

While direct evidence of RSV’s effects on macrophage activity in prostate cancer is limited, evidence of RSV’s effects on macrophages in other cancers is well documented. Based on their roles in tumor development, the macrophages in the tumor tissues can be divided into either M1- or M2-like subtypes. The M1-like macrophages possess an antitumor property by promoting Th1 response, and the M2-like macrophages usually promote Th2 response, tissue remodeling, immune tolerance, and tumor progression [123]. TAMs are phenotypically similar to M2 macrophages [124]. Recently, Kimura et al. experimentally demonstrated that RSV can prevent tumor cell metastasis by inhibiting M2-like macrophage differentiation [125]. Consistent with the findings that prostate cancer can secret certain factors, including IL-10, to induce M2 polarization [126], the authors further demonstrated that RSV is capable of inhibiting M2 differentiation by downregulating IL-10 secretion. Finally, oral administration of RSV (25 mg/kg, twice daily) for 30 days to mice injected with the highly metastatic osteosarcoma LM8 cells significantly reduced the number of metastatic tumors in both the lungs and the liver. These in vitro and in vivo data altogether unambiguously demonstrate that RSV is indeed capable of inhibiting cancer cell metastasis. However, whether RVS has a similar effect on prostate cancer cell metastasis still needs to be determined experimentally.

## 5. Conclusions and Perspectives

Since its anti-inflammation and anti-oxidation activities were demonstrated experimentally in 1997 [22], RSV and its derivatives have attracted great attention in both basic science and clinical settings. Because of its beneficial effects on improved metabolism, cardio protection, and cancer prevention, RSV has even been widely accepted as an anti-aging reagent. The general beneficial effects of RSV on many diseases have been thoroughly reviewed by Baur and Sinclair [23]. Discussion of clinical literature, along with the limitations of preclinical and in vitro RSV studies, can be found in the review article by Tome-Carneiro et al. [127]. The in vivo effects of resveratrol on different cancers, including breast, colorectal, liver, pancreatic, and prostate cancer have been systematically reviewed by Lindsay G. Carter [128]. More importantly, data from both in vitro and in vivo experiments indicate that RSV is one of a few natural products with minimal adverse effects. These findings make RSV an ideal preventive and therapeutic reagent. 

Because of the ubiquitous role of the androgen/AR axis in each process of prostate cancer development, including initiation, progression, and metastasis, special attention has been paid to the effects of RSV on AR. Relatively little attention has been paid to the RSV effect on epithelium–stroma interaction, although it is well known that RSV can exert its effects on prostate cancer in an AR-independent manner [129]. RSV has differential effects on growth, cell cycle arrest, and induction of apoptosis by RSV in human prostate cancer cell lines [130,131]. Overwhelming evidence suggests that RSV’s anti-prostate cancer effect is through targeting the AR and subsequent AR-regulated genes [50,132,133]. Harada et al. reported that RSV represses AR target gene expression, at least partially, by enhancing AR degradation in a time- and dose-dependent manner [134]. Along with other groups, we have demonstrated that RSV can repress AR function by inhibiting AR transcriptional activity [135]. In addition, a group of ~20 AR variants (ARVs) has been identified in prostate cancer, especially the metastatic castration-resistant prostate cancer (mCRPC). Among these variants, ARV7 has attracted special attention because of its androgen-independent transcriptional activity [136]. More recent findings suggest that approximately 10–30% of men with mCRPC are positive for AR-V7 [137]. We have also demonstrated that RSV is capable of repressing mCRPC cell growth by enhancing ARV7 poly-ubiquitination and subsequent degradation [138]. These findings suggest that resveratrol could be used as a prostate cancer preventive or therapeutic reagent.

Given the emerging roles of the stromal cells in cancer initiation and progression, great attention has been paid to the communication between stromal and cancer cells in recent years. Mounting evidence indicates that the stromal cells play indispensable roles in prostate cancer initiation, proliferation, and metastasis. As we have discussed in this review, by interrupting the epithelial and stromal cell communication, RSV can inhibit prostate cancer development through different mechanisms. However, there are still many unanswered questions about how RSV affects stromal–cancer cell communication. For example, it is well established that AR is expressed and functional in both epithelial and stromal cells [11]. But compared to our understanding of RSV’s effect on epithelial cells, very little is known about the role of RSV in stromal cells. Besides, ARV7 plays an indispensable role in mCRPC, and resveratrol can inhibit the proliferation of AVR7-positive cells. Our unpublished data indicate that RSV can affect the E-cadherin and N-cadherin ratio in ARV7-positive prostate cancer cells, suggesting that RSV could potentially be able to affect the metastasis of these cells. Whether ARV7 is simultaneously or subsequently expressed in cancer and stromal cells is unknown. If ARV7 is indeed expressed in stromal cells, will RSV also affect the stromal cells through targeting ARV7 as it did in the cancer cells? Answers to these questions and a better understanding of the underlying molecular mechanisms in how RSV inhibits prostate cancer progression by interrupting stromal–cancer cell communication will pave the road toward the application of RSV as both a cancer preventive and therapeutic reagent.
